# Clinical impact of number of lymph nodes dissected on postoperative survival in node-negative small cell lung cancer

**DOI:** 10.3389/fonc.2022.962282

**Published:** 2022-11-21

**Authors:** Shinkichi Takamori, Takefumi Komiya, Emily Powell

**Affiliations:** ^1^ Department of Surgery and Science, Graduate School of Medical Sciences, Kyushu University, Fukuoka, Japan; ^2^ Medical Oncology, Parkview Cancer Institute, Fort Wayne, IN, United States; ^3^ Division of Hematology Oncology, University at Buffalo, Buffalo, NY, United States; ^4^ Parkview Research Center, Mirro Center for Research and Innovation, Fort Wayne, IN, United States; ^5^ Oncology Research Program, Parkview Cancer Institute, Fort Wayne, IN, United States

**Keywords:** cancer, prognosis, lung small cell lung cancer, lymph node dissection, surgery, survival

## Abstract

**Objectives:**

Small cell lung cancer (SCLC) is a lethal histologic subtype of lung cancer. Although the Commission on Cancer recommends pathological examination of at least 10 lymph nodes dissected (LNDs) for resected early-stage non-small cell lung cancer, its survival benefit of LNDs in patients with early-stage SCLC is unknown.

**Methods:**

The National Cancer Database was queried for SCLC patients with clinical stage I-II and clinical N0, NX disease per AJCC 7^th^ edition who had undergone lobectomy between 2004 and 2017. Overall survival of SCLC patients by the number of LNDs was compared using Log-rank tests. Univariate and multivariable Cox proportional hazards analyses were performed.

**Results:**

In total, 688 (42%), 311 (20%), 247 (16%), 196 (12%), 126 (8%), and 36 (2%) of 1,584 patients with early-stage SCLC had ≥10, 7-9, 5-6, 3-4, 1-2, and 0 LNDs, respectively. The sequential improvement in the HRs was no longer evident if the number of LNDs exceeds 4. Patients with ≥3 LNDs (n = 1,422) had a significantly longer overall survival than those with <3 LNDs (n = 162) (hazard ratio for death: 0.76, 95% confidence interval: 0.62–0.94, *P* = 0.0087). Multivariate analysis revealed that ≥3 LNDs was an independent factor for predicting overall survival (hazard ratio for death: 0.76, 95% confidence interval: 0.61–0.93, *P* = 0.0083).

**Conclusions:**

Although we are reluctant to recommend a definitive “optimal number” of LNDs, our findings suggest the prognostic and therapeutic roles for performing ≥3 LNDs in patients with early-stage SCLC who undergo lobectomy.

## Introduction

Lung cancer is one of the most fatal malignancies worldwide (1). The standard therapy for resectable lung cancer is lobectomy and thoracic lymphadenectomy (2, 3). The majority of lung cancers are classified as non-small cell lung cancer (NSCLC), and therefore the majority of studies have centered around this histologic subtype. With regard to patients with early-stage NSCLC, the required extent of thoracic lymphadenectomy has been debated (4–8). Several previous studies reported that systemic lymph node (LN) dissection provided a longer disease-free survival and overall survival (OS) than mediastinal LN sampling in patients with early-stage NSCLC (5, 6). National Comprehensive Cancer Network (NCCN) guidelines advocate sampling of at least three N2 stations or a complete mediastinal dissection (9). The Commission on Cancer (CoC) recommends that at least 10 LNs should be pathologically examined for resected early-stage NSCLC (10, 11).

Small cell lung cancer (SCLC) is the most lethal histologic subtype of lung cancer for which there have been small advances in treatment over the past decade (12). For early-stage SCLC, the use of surgery is recommended based on retrospective or single arm studies (13, 14). The International Association for the Study of Lung Cancer database for the 7th editions of the International Staging System showed that SCLC patients with clinical T1a disease who underwent surgery had 93% survival at 12 months and 73% at 24 months. SCLC patients with clinical T1b disease who received surgery had 89% survival at 12 months and 76% at 24 months (15). In general, SCLC patients who are candidates for surgery are rare, since most patients with SCLC present with locally advanced or distant metastases (12). Due to the rarity of SCLC patients who are candidates for surgery, the required extent of thoracic lymphadenectomy for early-stage SCLC has not been comprehensively investigated. The aim of the current study is to examine the prognostic significance of the number of LNs dissected (LNDs) in patients with early-stage SCLC who receive curative lung resection.

## Materials and methods

### National cancer database (NCDB)

The NCDB is a joint project between the CoC of the American College of Surgeons and the American Cancer Society. The CoC’s NCDB and the hospitals participating in the CoC NCDB are the source of the de-identified data used herein; they have not verified and are not responsible for the statistical validity of the data analysis or the conclusions derived by the authors. The data is considered as hospital-based rather than population-based (16). The access to the NCDB participant use file was granted to T.K. Based on the use of only de-identified data, the study was exempted by the Parkview institutional review board.

**Figure 1:**
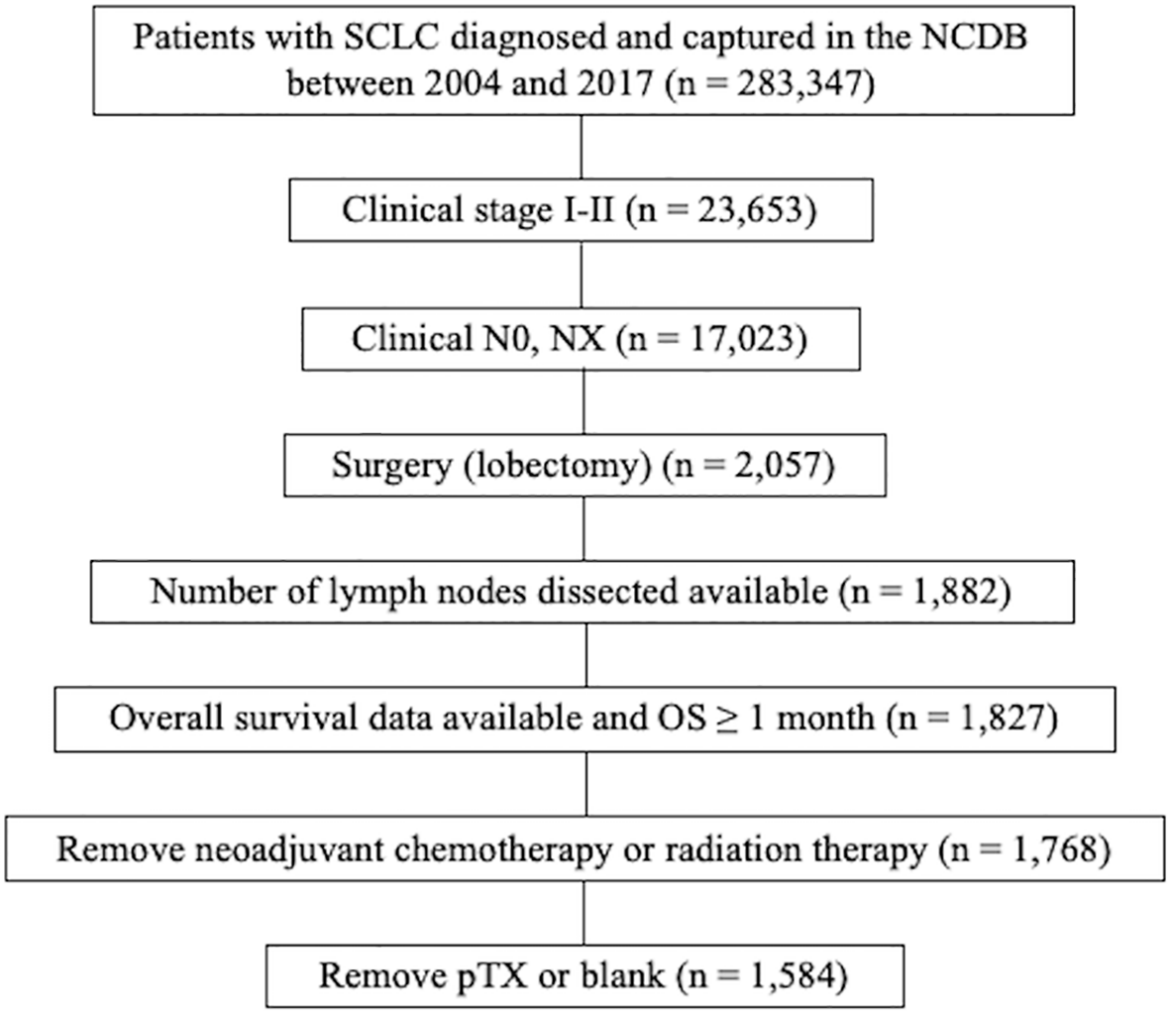
Study flow diagram of case eligibility. SCLC, small cell lung cancer; NCDB, National Cancer Database; AJCC, American Joint commission on cancer; OS, overall survival.

Patients with SCLC diagnosed and captured in the NCDB between 2004 and 2017 were selected (n = 283,347). Of these, patients with clinical stage I-II disease were included (n = 23,653). Patients with clinical N0 and NX disease were selected (n = 17,023). Patients who underwent surgery (lobectomy) were then selected (n = 2,057). Of these, patients with information about number of lymph nodes dissected were included (n = 1,882). Patients whose survival data were available and who survived at least 1 month past the date of diagnosis were then selected (n = 1,827). Patients with neoadjuvant chemotherapy or radiation therapy were excluded (n = 1,768). Of these, patients with pTX or blank were excluded (n = 1,584). The study flow diagram of case eligibility is shown in **Figure 1**.

Clinical demographics including age (<70 vs. 70+), sex (male vs. female), race (whites vs. others), insurance (insured vs. uninsured), institutions (academic vs. others), Charlson-Deyo comorbidity score (0–1 vs. ≥2), years of diagnosis (2004-2010 vs. 2011-2017), histology (SCLC, not otherwise specified [NOS] vs. others), pathologic T stage (T0-1 vs. T2-4), pathologic N stage (N0 vs. N1-2), tumor size (<30 mm vs. ≥30 mm), resected margin status (other vs. negative), adjuvant chemotherapy (yes vs. no/unknown), and adjuvant chest radiation (yes vs. no/unknown) were collected. We chose the median year (2010) as a cut-off for breaking up the years of diagnosis based on the previous report, indicating that the mortality from SCLC has been declining in a linear fashion (17).

## Statistical analysis

Kaplan-Meier curves by the number of LNDs were compared using the log-rank test. The associations between the number of LNDs and clinical demographics were assessed by chi-squared test and Fisher’s two-sided exact test where appropriate. Univariate and multivariable Cox proportional hazards analyses were performed using JMP^®^ 14.0 (SAS Institute Inc., Cary, NC, USA). A two tailed, *p* < 0.05 was considered statistically significant.

## Results

### Patient characteristics

The study flow diagram of case eligibility is shown in **Figure 1**. Of note, this study analyzed only SCLC patients who received lobectomy. Patient characteristics (n = 1,584) are summarized in **Table 1**. In total, 688 (42%), 85 (5%), 112 (7%), 114 (7%), 110 (7%), 137 (9%), 105 (7%), 91 (6%), 87 (6%), 39 (2%), and 36 (2%) of 1,584 patients with early-stage SCLC had ≥10, 9, 8, 7, 6, 5, 4, 3, 2, 1, and 0 LNDs, respectively. As shown in **Table 2**, patients with ≥3 LNDs were significantly associated with insured (P = 0.0194) and years of diagnosis (P = 0.0263) per univariate analysis. In patients with cN0/pN1-2 disease (n = 311), 295 (95%) had LNDs≥3, and 16 (5%) had LNDs<3. In patients with cN0/pN0 disease (n = 1,246), 1,122 (90%) had LNDs≥3, and 124 (10%) had LNDs<3. Significantly more patients in the cN0/pN1-2 group received LNDs≥3 than in the cN0/pN0 group (P = 0.0075). With the aim of analyzing the effect of LNDs on nodal upstaging, pN+ rates ([pN+ cases] divided by [pN+ cases + pN0 cases]) according to the number of LNDs were calculated (**Supplementary Figure 1**). The sequential increase in the nodal upstaging was suggested if the number of LNDs increased.

**Table 1:** Patient characteristics of resected clinical stage I-II (AJCCv7) small cell lung cancer (n = 1,584).

Factors		Value or no. of patients
Age	<70	947 (60%)
	≥70	637 (40%)
Sex	male	713 (45%)
	female	871 (55%)
Race	whites	1,449 (91%)
	others	135 (9%)
Insurance status	uninsured	22 (1%)
	insured	1,562 (99%)
Institution	academic	601 (40%)
	others	983 (60%)
Charlson-Deyo score	0-1	1,336 (84%)
	≥2	248 (16%)
Year of diagnosis	2004-2010	404 (26%)
	2011-2017	1,180 (74%)
Histology	SCLC NOS	1,191 (75%)
	others	393 (25%)
Pathologic T stage	T0-1	963 (61%)
	T2-4	621 (39%)
Pathologic N stage	N0	1,246 (79%)
	N1-2	311 (19%)
	NX	27 (2%)
Tumor size	<30mm	493 (31%)
	≥30mm	1,091 (69%)
Resected margin status	other	76 (5%)
	negative	1,508 (95%)
Adjuvant chemotherapy	yes	1,052 (66%)
	multiagent chemotherapy	976 (61%)
	single agent chemotherapy	29 (2%)
	unknown	47 (3%)
	no	477 (30%)
	unknown	55 (4%)
Adjuvant chest radiation	yes	423 (27%)
	no/unknown	1,161 (73%)
Number of lymph nodes dissected	0	36 (2%)
	1	39 (2%)
	2	87 (6%)
	3	91 (6%)
	4	105 (7%)
	5	137 (9%)
	6	110 (7%)
	7	114 (7%)
	8	112 (7%)
	9	85 (5%)
	≥10	668 (42%)

AJCC, American Joint Commission on Cancer; SCLC, small cell lung cancer; NOS, not otherwise specified.

**Table 2:** Patient characteristics of resected clinical stage I-II (AJCCv7) small cell lung cancer according to number of lymph nodes dissected (n = 1,584).

Factors		Number of lymph nodes dissected		*P* value
		≥3 (n = 1,422)	<3 (n = 162)	
Age	<70	847 (60%)	100 (62%)	0.5945^a^
	≥70	575 (40%)	62 (38%)	
Sex	male	643 (45%)	70 (43%)	0.6264^a^
	female	779 (55%)	92 (57%)	
Race	whites	1,301 (91%)	148 (91%)	0.9542^a^
	others	121 (9%)	14 (9%)	
Insurance status	uninsured	X	X	0.0194^b^
	insured	Y	Y	
Institution	academic	547 (38%)	54 (33%)	0.2020^a^
	others	875 (62%)	108 (67%)	
Charlson-Deyo score	0-1	1,200 (84%)	136 (84%)	0.8845^a^
	≥2	222 (16%)	26 (16%)	
Year of diagnosis	2004-2010	351 (25%)	53 (33%)	0.0263^a^
	2011-2017	1,071 (75%)	109 (67%)	
Histology	SCLC NOS	1,060 (75%)	131 (81%)	0.0776^a^
	others	362 (25%)	31 (19%)	
Pathologic T stage	T0-1	863 (61%)	100 (62%)	0.7974^a^
	T2-4	559 (39%)	62 (38%)	
Pathologic N stage*	N0	1,122 (79%)	124 (89%)	0.0075^b^
	N1-2	295 (21%)	16 (11%)	
Tumor size	<30mm	441 (31%)	52 (32%)	0.7773^a^
	≥30mm	981 (69%)	110 (68%)	
Resected margin status	other	65 (5%)	11 (7%)	0.2105^a^
	negative	1,357 (95%)	151 (93%)	
Adjuvant chemotherapy	yes	908 (64%)	97 (60%)	0.3193^a^
	no/unknown	514 (36%)	65 (40%)	
Adjuvant chest radiation	yes	380 (27%)	43 (27%)	0.9609^b^
	no/unknown	1,042 (73%)	119 (73%)	

AJCC, American Joint Commission on Cancer; SCLC, small cell lung cancer; NOS, not otherwise specified, X and Y number less than 10 cannot be reported according to NCDB agreement.

a χ^2^ test.

b Fisher’s 2-sided exact test.

*cases with pathologic NX (n = 37) were excluded.

### Univariate survival analyses in patients with early-stage SCLC according to the number of LNs dissected

**Figure 2:**
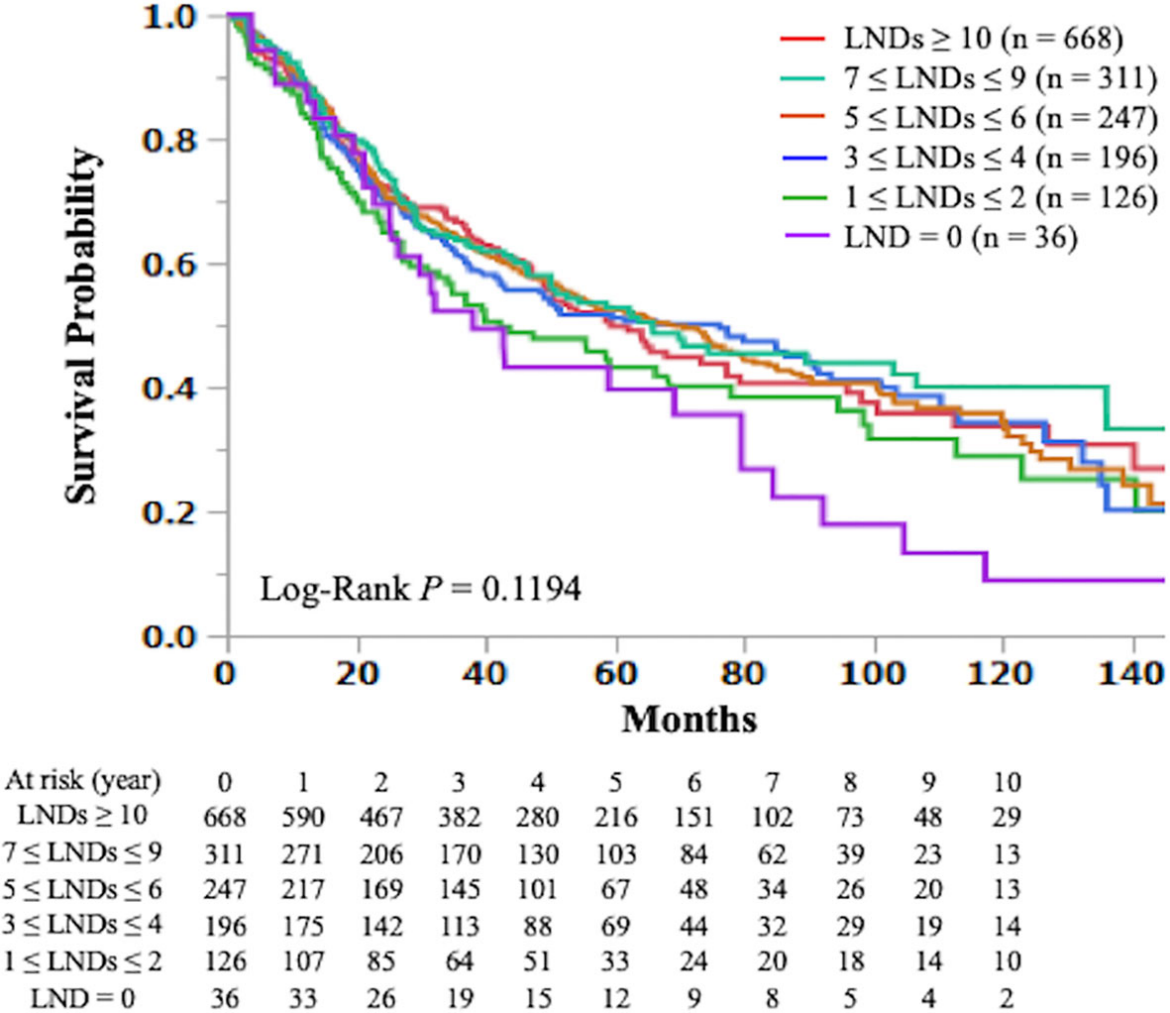
The Kaplan-Meier curves of overall survival in early-stage small cell lung cancer patients who underwent curative lobectomy according to the number of lymph nodes dissected (≥ 10 vs. 7-9 vs. 5-6 vs. 3-4 vs. 1-2 vs. 0) are shown. LND; lymph node dissected.

**Figure 3:**
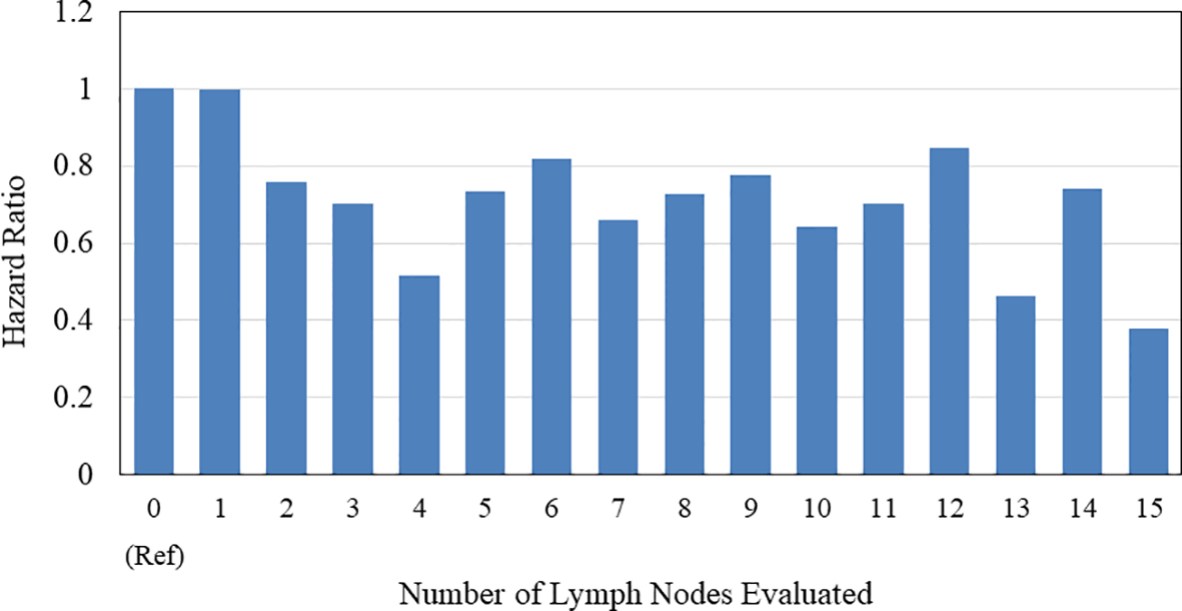
Hazard ratios for death by the number of lymph nodes evaluated are shown.

**Figure 4:**
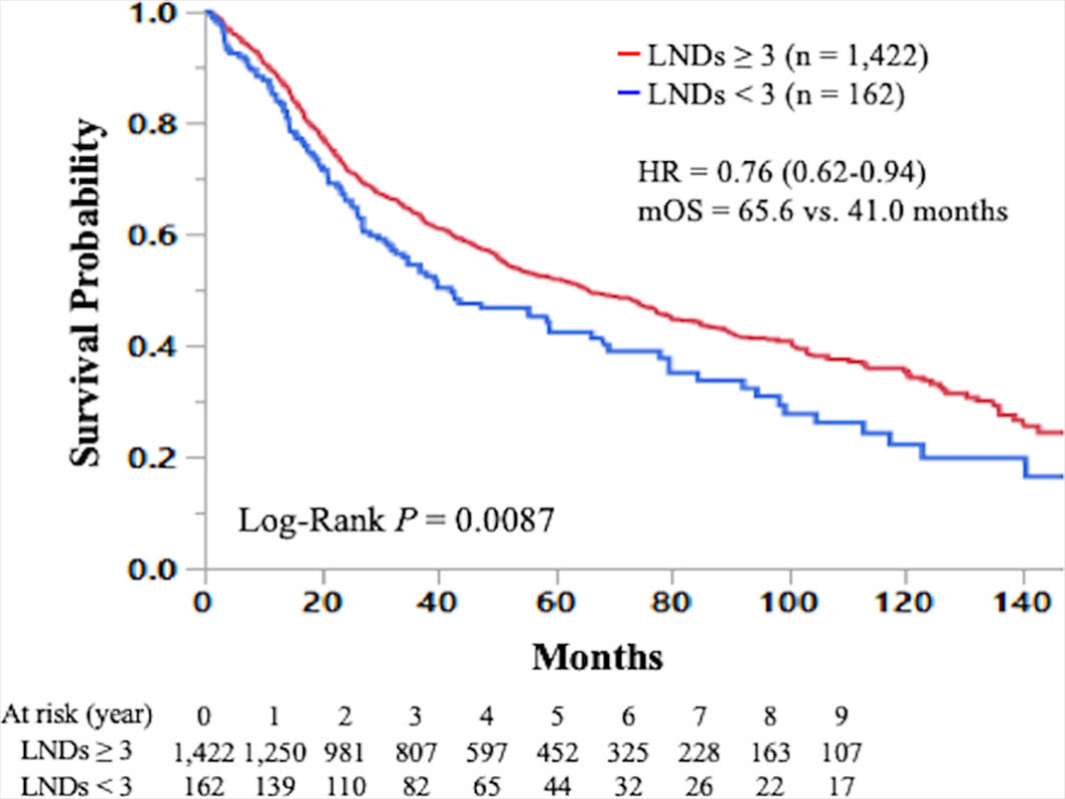
Kaplan-Meier curve of overall survival in early-stage small cell lung cancer patients who underwent curative lobectomy according to the number of lymph nodes dissected (≥3 vs. <3) is shown. LND; lymph node dissected; HR, hazard ratio; mOS, median overall survival.

The Kaplan-Meier curve comparing OS according to the number of LNDs in patients with early-stage SCLC is shown in **Figure 2**. The OS was not significantly influenced by the number of LNDs (P = 0.1194). Multivariable COX regression analysis in subgroup-by-subgroup comparisons according to LNDs was performed to find the appropriate cut point. **Figure 3** shows the hazard ratios [HR] for death by the number of LNDs. The sequential improvement in the HRs was no longer evident if the number of LNDs exceeds 4. A minimum of 3 LNs evaluation was needed to improve the mortality risk over compared with that without LNDs. As shown in **Figure 4**, patients with ≥3 LNDs had a significantly longer OS than those with <3 LNDs (median OS: 65.6 vs. 41.0 months, HR for death: 0.76, 95% confidence interval [CI]: 0.62–0.94, P = 0.0087). Since the NCCN guidelines recommend surgery for selected patients with T1-2/N0 SCLC and consider it for some patients with T3/N0 SCLC while surgery is not recommended for T4/N0 disease if invasive mediastinal lymph node staging is negative, a prognosis subgroups analysis stratified by T stage was conducted. In subgroup analysis of patients with T0-2 disease, patients with ≥3 LNDs had a significantly longer OS than those with <3 LNDs (P = 0.0006; **Supplementary Figure 2A**). However, in subgroup analysis of patients with T3-4 disease, OS of patients with ≥3 LNDs was similar to that of patients with <3 LNDs (P = 0.9968; **Supplementary Figure 2B**). From **Figure 2**, it can be seen that only the LND = 0 group had the worst OS and separated from the others. Therefore, we subsequently excluded the LND = 0 group, and analyzed the OS data. Patients with ≥3 LNDs tended to have a longer OS than those with LNDs = 1, 2 (P = 0.0683; **Supplementary Figure 3**). In addition, patients with ≥10 LNDs did not have a significantly longer OS than those with <10 LNDs (HR for death: 0.95, 95% CI: 0.82–1.09, P = 0.6296, **Supplementary Figure 4**). The subgroup analysis of cN0/pN0 patients showed that patients with ≥3 LNDs had a significantly longer OS than those with <3 LNDs (P = 0.0041; **Supplementary Figure 5**). The survival curve comparing OS according to the number of LNDs is shown in **Supplementary Figure 6**. The OS was significantly influenced by the number of LNDs (P = 0.0178).

### Univariate and multivariable analyses of OS in early-stage SCLC patients who underwent curative lobectomy

The results of univariate and multivariable analyses for OS in early-stage SCLC patients are shown in **Table 3**. Univariate analysis showed that age (*P* < 0.0001), sex (*P* = 0.0015), Charlson-Deyo score (*P* = 0.0018), pathologic T stage (*P* < 0.0001), pathologic N stage (*P* < 0.0001), tumor size (*P* = 0.0055), resected margin status (*P* = 0.0114), adjuvant chemotherapy (*P* = 0.0004), and ≥3 LNDs (HR for death: 0.76, 95% CI: 0.62–0.94, *P* = 0.0115) were significantly associated with OS. In multivariable analysis, age (*P* < 0.0001), sex (*P* = 0.0039), Charlson-Deyo score (*P* = 0.0063), pathologic T stage (*P* < 0.0001), pathologic N stage (*P* < 0.0001), resected margin status (*P* = 0.0341), adjuvant chemotherapy (*P* < 0.0001), and ≥3 LNDs (HR for death: 0.76, 95% CI: 0.61–0.93, *P* = 0.00083) were independent factors for predicting OS. In the subgroup multivariate analysis of OS in the cN0/pN1-2 group (n = 311) showed that LNDs≥3 was an independent prognostic factor (HR for death: 0.52, 95% CI: 0.30–0.96, *P* = 0.0372; **Supplementary Table 1**).

**Table 3:** Multivariable analyses of overall survival in patients with resected clinical stage I-II (AJCCv7) small cell lung cancer.

Factors		Univariate	Multivariable
		HR (95% CI)	HR (95% CI)
		*P* value	*P* value
Age	<70	0.67 (0.59-0.77)	0.70 (0.60-0.80)
	≥70 (Ref)	< 0.0001	< 0.0001
Sex	female	0.80 (0.70-0.92)	0.81 (0.71-0.94)
	male (Ref)	0.0015	0.0039
Race	whites	1.01 (0.80-1.30)	0.94 (0.74-1.22)
	others (Ref)	0.9398	0.6421
Insurance status	uninsured	0.68 (0.32-1.23)	0.67 (0.32-1.22)
	others (Ref)	0.2166	0.2085
Institution	academic	1.05 (0.91-1.21)	0.96 (0.83-1.11)
	others (Ref)	0.4994	0.6215
Charlson-Deyo score	0-1	0.75 (0.63-0.89)	0.77 (0.65-0.93)
	≥2 (Ref)	0.0018	0.0063
Year of diagnosis	2004-2010	0.87 (0.74-1.02)	0.86 (0.73-1.00)
	2011-2017 (Ref)	0.0840	0.0602
Histology	others	1.05 (0.89-1.22)	1.01 (0.86-1.19)
	SCLC NOS (Ref)	0.5787	0.8631
Pathologic T stage	T0-1	0.67 (0.58-0.76)	0.69 (0.58-0.83)
	T2-4 (Ref)	<0.0001	< 0.0001
Pathologic N stage	N0	0.45 (0.39-0.52)	0.44 (0.38-0.51)
	N1-2/NX (Ref)	< 0.0001	< 0.0001
Tumor size	<30mm	0.81 (0.70-0.94)	1.09 (0.90-1.32)
	≥30mm (Ref)	0.0055	0.3864
Resected margin status	negative	0.68 (0.52-0.91)	0.73 (0.55-0.98)
	other (Ref)	0.0114	0.0341
Adjuvant chemotherapy	yes	0.77 (0.67-0.89)	0.73 (0.62-0.85)
	no (Ref)	0.0004	< 0.0001
Adjuvant chest radiation	no/unknown	1.02 (0.88-1.19)	0.97 (0.81-1.15)
	yes (Ref)	0.8022	0.6959
Number of lymph nodes dissected	3≥	0.76 (0.62-0.94)	0.76 (0.61-0.93)
	<3 (Ref)	0.0115	0.0083

AJCC, American Joint Commission on Cancer; SCLC, small cell lung cancer; NOS, not otherwise specified; Ref, reference.

## Discussion

In the current study, we reported for the first time that SCLC patients with ≥3 LNDs had a significantly longer OS than those with <3 LNDs. The multivariate analysis showed that ≥3 LNDs was an independent predictor for OS. Of note, the HR for death was 0.75 in patients with ≥3 LNDs compared with those with <3 LNDs, which suggests its significant prognostic and therapeutic impact. Further analyses showed that the difference in OS was not significant for cut-off of 10 LNDs. Given that the CoC recommends pathological examination of at least 10 LNs for resected early-stage NSCLC (10, 11), the appropriate cut-off for the minimal number of LNDs in early-stage SCLC may be less than that in NSCLC. Although we are reluctant to recommend a definitive “optimal number” of LNs evaluated, our findings suggested the prognostic and therapeutic roles for performing ≥3 LNDs in patients with resectable SCLC.

The recommended number of surgical LNDs for early-stage SCLC has never been investigated in the past clinical trials. This is due to the rarity of early-stage SCLC patients who are candidates for surgery. According to the previous report, stage I disease accounts for less than 5% of patients with SCLC, and patients with disease in excess of T1-2, N0 did not benefit from surgery (18). Given that highly selected SCLC patients are candidate for surgery, future randomized trials investigating the required extent of thoracic lymphadenectomy for early-stage SCLC may not be possible. Although our study was a retrospective study, the largest cancer database enrolled a total of 1,584 patients with resected SCLC, and suggested that at least 3 LNDs is recommended for early-stage SCLC.

We consider that survival gain resulting from LNDs is due to both diagnostic and therapeutic roles of LNDs. Regarding diagnostic role, the high-quality LNDs allow for accurate stage migration, subsequently optimal postoperative treatment, and improves patients’ prognosis (16). Pathological nodal upstaging cases are identified in 10-20% of patients with clinical node-negative NSCLC (19). We presumed that the benefit from more LNDs was more accurate detection of nodal involvement in cN0 patients. Therefore, we conducted additional analysis regarding pN+ rates ([pN+ cases] divided by [pN+ cases + pN0 cases]) as shown in **Supplementary Figure 1**. As the number of LNDs increased, the upstaging rate of nodal status sequentially increased, which suggests that the high-quality LNDs enables accurate nodal staging. According to the NCCN guideline, postoperative chest radiation therapy is indicated for the patients with node-positive SCLC (9). With regard to therapeutic role, adequate LNDs can remove any remaining metastatic LNs and increase the cure rate. According to the previous report, the number of LNDs less than or equal to 15 was an independent predictor of higher probability of local recurrence in patients with completely resected pathological stage I NSCLC (20).

There are several limitations in association with our study. First, NCDB databases lack the data regarding the LN stations of the LNs investigated, which has been reported to be associated with OS in patients with NSCLC (21–24). The anatomical location of the positive LN stations has a significant effect on the prognostic value of the proportion of positive LNs (25). Second, the number of LNs removed is influenced by surgeon and pathologist procedures. Regarding surgeon procedure, if some LNs are removed in fragments, as often occurs during lung cancer resections, the pathologist can end up identifying a greater number of total LNs. From the standpoint of the pathologist, failure to remove and examine pathologically the peribronchial LNs that are removed but not separately labeled by the surgeon during a lobectomy can lead to failure of identifying N1 LN involvement (26–29) Third, this is a retrospective study in association with a bias from surgeon’s decisions. Surgeons may take more LNs in the middle of surgery if the LNs look suspicious of metastases. However, our study showed that patients with ≥3 LNDs had a significantly longer OS than those with fewer LNDs. Thus, the bias arising from surgeons’ choice may not significantly contribute to longer OS in patients with fewer LNDs in the current study. Fourth, NCDB is lacking in the information about how surveillance was conducted, how patients were staged preoperatively, adjuvant treatment, operative time, and center-level effects. Further advanced study is needed to reach the definitive conclusions.

In conclusion, our retrospective analysis using the largest cancer database showed for the first time that patients with ≥3 LNDs had a significantly longer OS than those who had undergone fewer LNDs, suggesting prognostic and therapeutic roles for performing ≥3 LNDs. Further research is warranted to validate these findings.

## Data availability statement

The original contributions presented in the study are included in the article/**Supplementary Materials**. Further inquiries can be directed to the corresponding author.

## Ethics statement

Based on the use of only de-identified data, the study was exempted by the Parkview institutional review board. Written informed consent from the participants’ legal guardian/next of kin was not required to participate in this study in accordance with the national legislation and the institutional requirements.

## Author contributions

ST contributed to the interpretation of data, and wrote the manuscript. TK contributed to all of the ideas of the current study and methods of analyzing the data. EP supervised the writing of the manuscript. All authors significantly contributed to this study. All authors read and approved the final manuscript.

## Acknowledgments

We thank Mindy Flannagan and Mototsugu Shimokawa for statistical assistance.

## Conflict of interest

TK received travel fee from Merck.

The remaining authors declare that the research was conducted in the absence of any commercial or financial relationships that could be construed as a potential conflict of interest.

## Publisher’s note

All claims expressed in this article are solely those of the authors and do not necessarily represent those of their affiliated organizations, or those of the publisher, the editors and the reviewers. Any product that may be evaluated in this article, or claim that may be made by its manufacturer, is not guaranteed or endorsed by the publisher.

## Supplementary material

Supplementary Figure 1 Pathological nodal positive rates ([pN+ cases] divided by [pN+ cases + pN0 cases]) according to the number of lymph nodes dissected (≥ 10 vs. 7-9 vs. 4-6 vs. 1-3) are shown.

Supplementary Figure 2 Kaplan-Meier curve of overall survival in **(A)** T0-2 and **(B)** T3-4 small cell lung cancer patients who underwent curative lobectomy according to the number of lymph nodes dissected (≥3 vs. <3) is shown. LND; lymph node dissected.

Supplementary Figure 3 The Kaplan-Meier curves of overall survival in early-stage small cell lung cancer patients with pN0 who underwent curative lobectomy according to the number of lymph nodes dissected (≥ 10 vs. 7-9 vs. 5-6 vs. 3-4 vs. 1-2 vs. 0) are shown. LND; lymph node dissected.
