# Supplementary material for: Clinical impact of number of lymph nodes dissected on postoperative survival in node-negative small cell lung cancer
Source: Front Oncol. 2022 Nov 21;12:962282. doi: 10.3389/fonc.2022.962282 (PMC9720149; doi:10.3389/fonc.2022.962282)
Supplement: Supplementary file 1 [file Table_1.docx]

**Supplementary Table 1**. Subgroup multivariable analyses of overall survival in patients with resected clinical stage I-II (AJCCv7) and cN0/pN1-2 small cell lung cancer (n = 311).

| **Factors** | | **Univariate** |  | **Multivariable** |
| --- | --- | --- | --- | --- |
|  |  | **HR (95% CI)** |  | **HR (95% CI)** |
|  |  | ***P* value** |  | ***P* value** |
| Age | <70 | 0.70 (0.53-0.90) |  | 0.68 (0.51-0.90) |
|  | ≥70 (Ref) | 0.0067 |  | 0.0084 |
|  |  |  | | |
| Sex | female | 0.90 (0.70-1.18) |  | 0.89 (0.68-1.18) |
|  | male (Ref) | 0.4518 |  | 0.4206 |
|  |  |  | | |
| Race | whites | 1.10 (0.70-1.85) |  | 0.95 (0.59-1.62) |
|  | others (Ref) | 0.6948 |  | 0.8480 |
|  |  |  | | |
| Insurance status | uninsured | 0.26 (0.04-0.81) |  | 0.31 (0.05-1.00) |
|  | others (Ref) | 0.0159 |  | 0.0506 |
|  |  |  | | |
| Institution | academic | 0.83 (0.63-1.08) |  | 0.77 (0.58-1.02) |
|  | others (Ref) | 0.1616 |  | 0.0655 |
|  |  |  | | |
| Charlson-Deyo score | 0-1 | 0.70 (0.51-0.98) |  | 0.73 (0.53-1.04) |
|  | ≥2 (Ref) | 0.0366 |  | 0.0779 |
|  |  |  | | |
| Year of diagnosis | 2004-2010 | 1.03 (0.76-1.39) |  | 0.92 (0.66-1.25) |
|  | 2011-2017 (Ref) | 0.8259 |  | 0.5851 |
|  |  |  | | |
| Histology | others | 1.20 (0.88-1.60) |  | 1.13 (0.83-1.53) |
|  | SCLC NOS (Ref) | 0.2424 |  | 0.4254 |
|  |  |  | | |
| Pathologic T stage | T0-1 | 0.79 (0.60-1.02) |  | 0.73 (0.53-1.02) |
|  | T2-4 (Ref) | 0.0698 |  | 0.0657 |
|  |  |  | | |
| Tumor size | <30mm | 0.99 (0.75-1.31) |  | 0.75 (0.53-1.06) |
|  | ≥30mm (Ref) | 0.9463 |  | 0.1007 |
|  |  |  | | |
| Resected margin status | negative | 0.57 (0.38-0.90) |  | 0.51 (0.33-0.83) |
|  | other (Ref) | 0.0172 |  | 0.0077 |
|  |  |  | | |
| Adjuvant chemotherapy | yes | 0.73 (0.55-0.99) |  | 0.69 (0.50-0.97) |
|  | no (Ref) | 0.0430 |  | 0.0341 |
|  |  |  | | |
| Adjuvant chest radiation | no/unknown | 1.20 (0.92-1.55) |  | 0.97 (0.71-1.31) |
|  | yes (Ref) | 0.1821 |  | 0.8533 |
|  |  |  | | |
| Number of lymph nodes dissected | 3≥ | 0.63 (0.38-1.14) |  | 0.52 (0.30-0.96) |
|  | <3 (Ref) | 0.1200 |  | 0.0372 |

AJCC, American Joint Commission on Cancer; SCLC, small cell lung cancer; NOS, not otherwise specified; Ref, reference.
