# Supplementary figures and images for: Clinical impact of number of lymph nodes dissected on postoperative survival in node-negative small cell lung cancer
Source: Front Oncol. 2022 Nov 21;12:962282. doi: 10.3389/fonc.2022.962282 (PMC9720149; doi:10.3389/fonc.2022.962282)

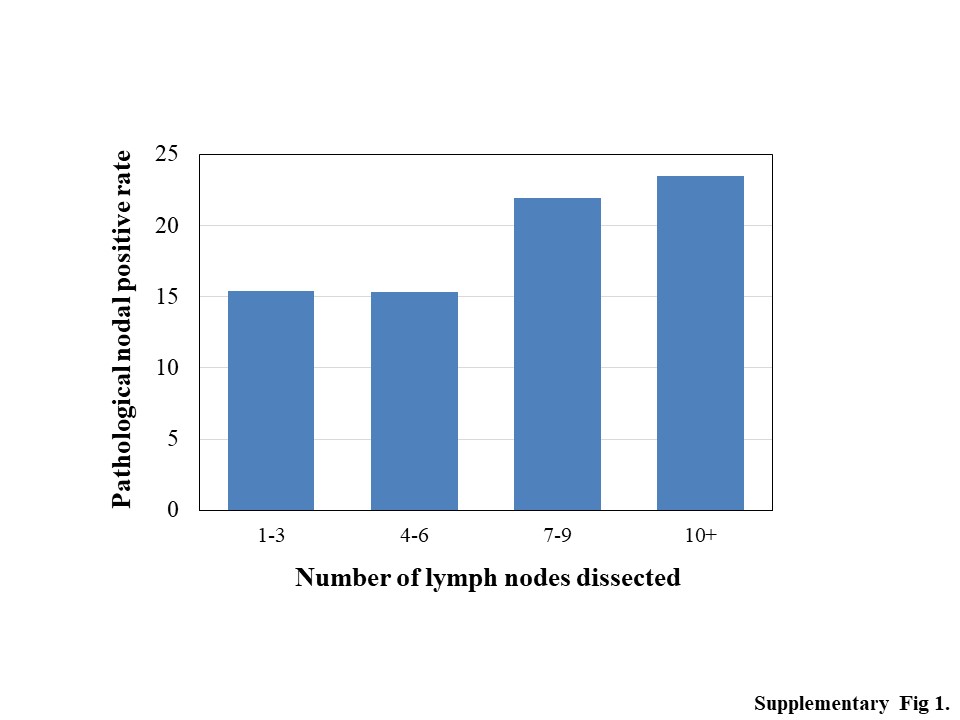

Supplement: Supplementary Figure 1 — Pathological nodal positive rates ([pN+ cases] divided by [pN+ cases + pN0 cases]) according to the number of lymph nodes dissected (≥ 10 vs. 7-9 vs. 4-6 vs. 1-3) are shown. [file Image_1.jpeg]

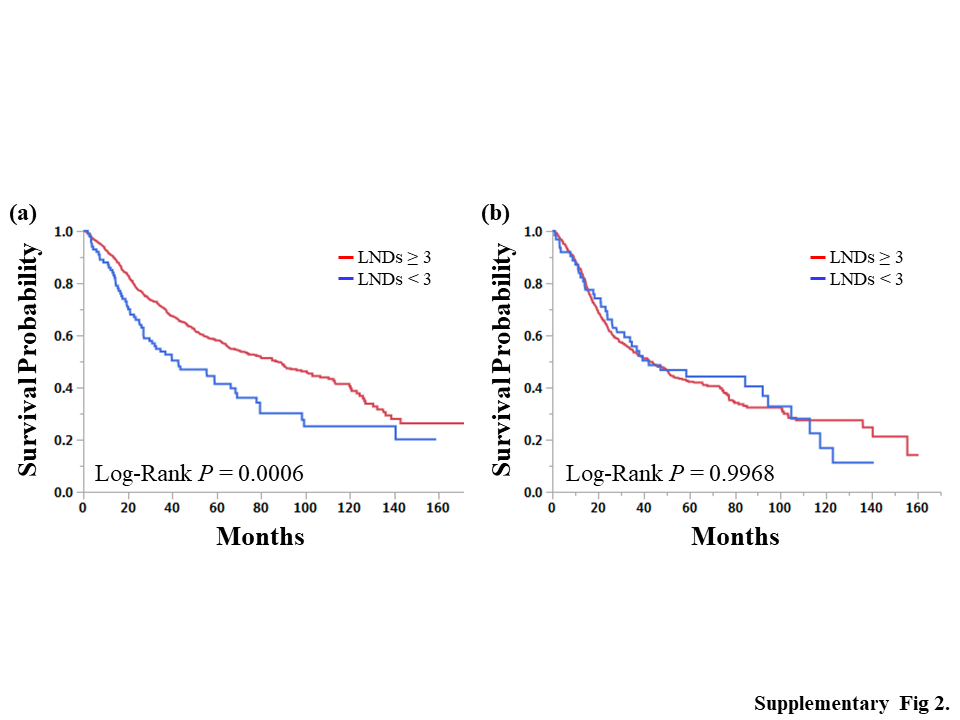

Supplement: Supplementary Figure 2 — Kaplan-Meier curve of overall survival in (A) T0-2 and (B) T3-4 small cell lung cancer patients who underwent curative lobectomy according to the number of lymph nodes dissected (≥3 vs. <3) is shown. LND; lymph node dissected. [file Image_2.tif]

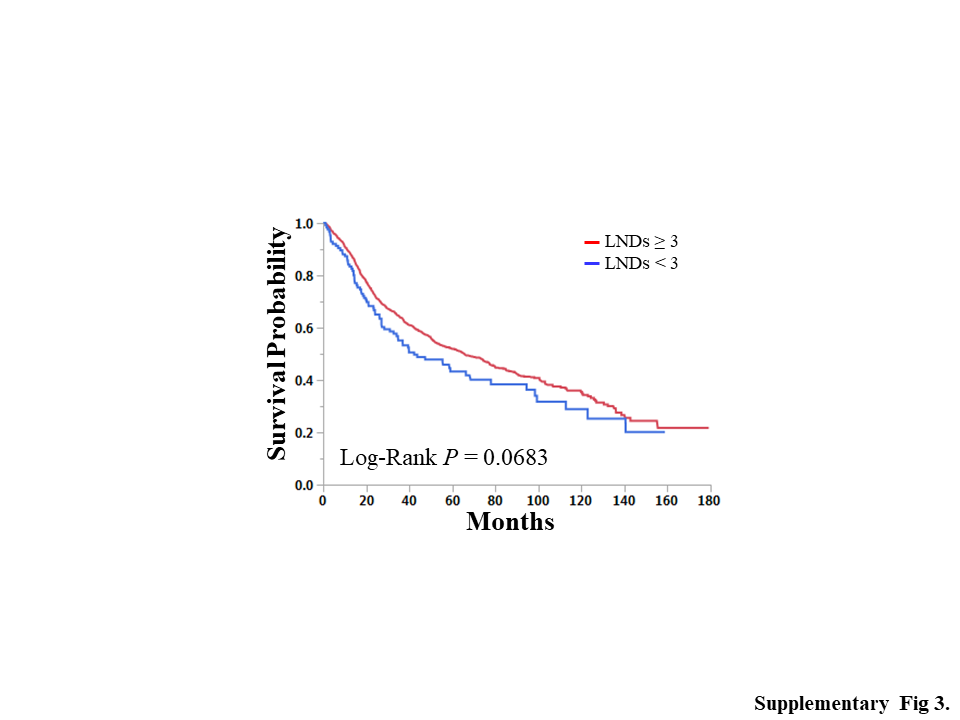

Supplement: Supplementary Figure 3 — The Kaplan-Meier curves of overall survival in early-stage small cell lung cancer patients with pN0 who underwent curative lobectomy according to the number of lymph nodes dissected (≥ 10 vs. 7-9 vs. 5-6 vs. 3-4 vs. 1-2 vs. 0) are shown. LND; lymph node dissected. [file Image_3.tif]

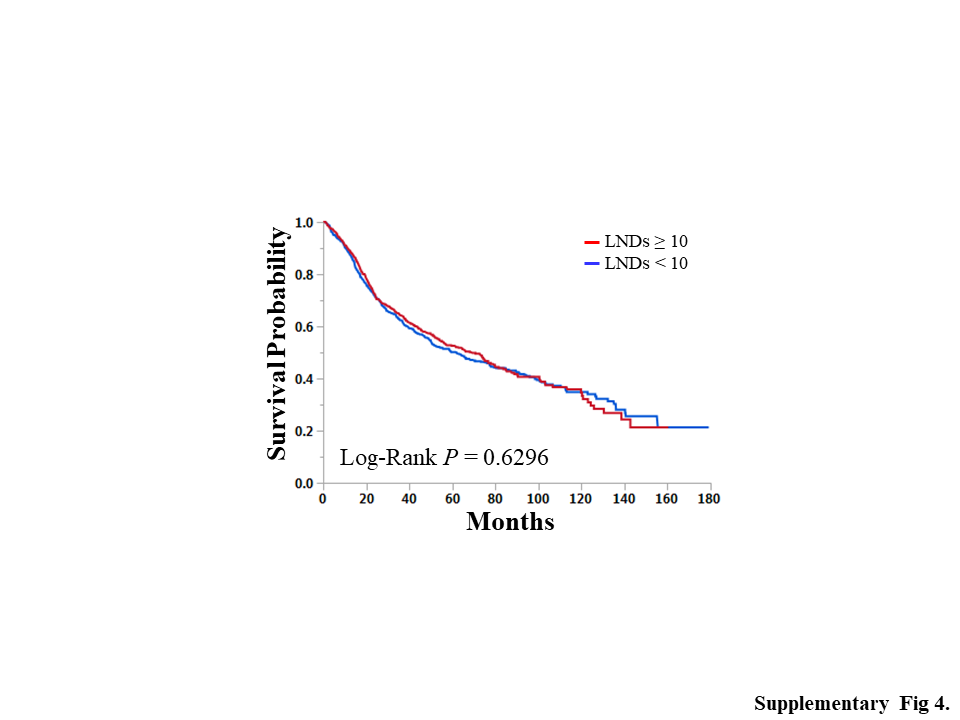

Supplement: Supplementary file 5 [file Image_4.tif]

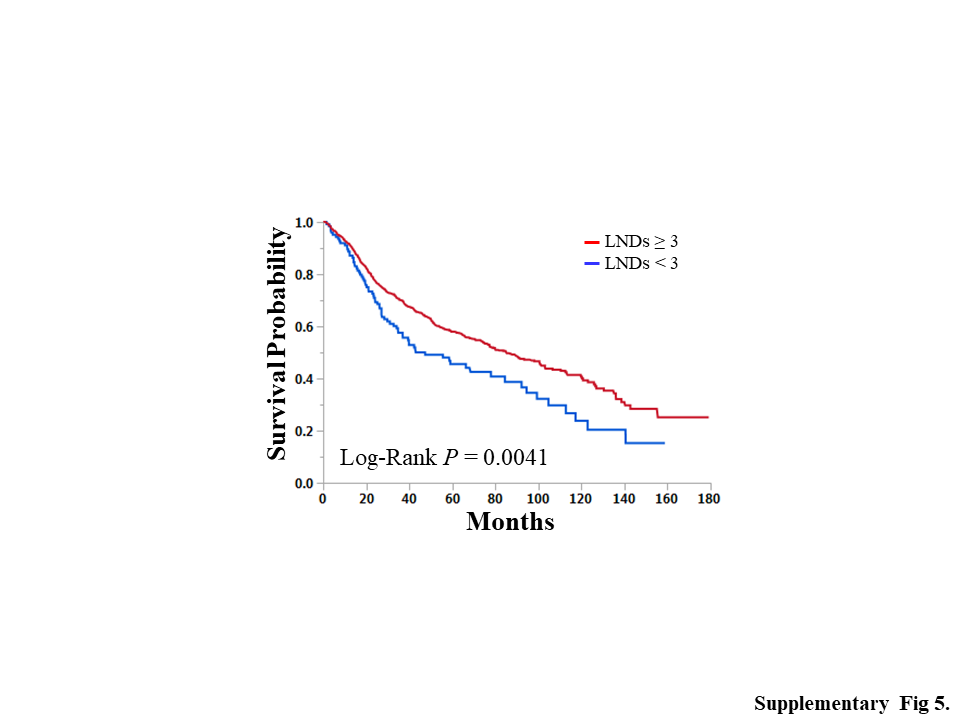

Supplement: Supplementary file 6 [file Image_5.tif]

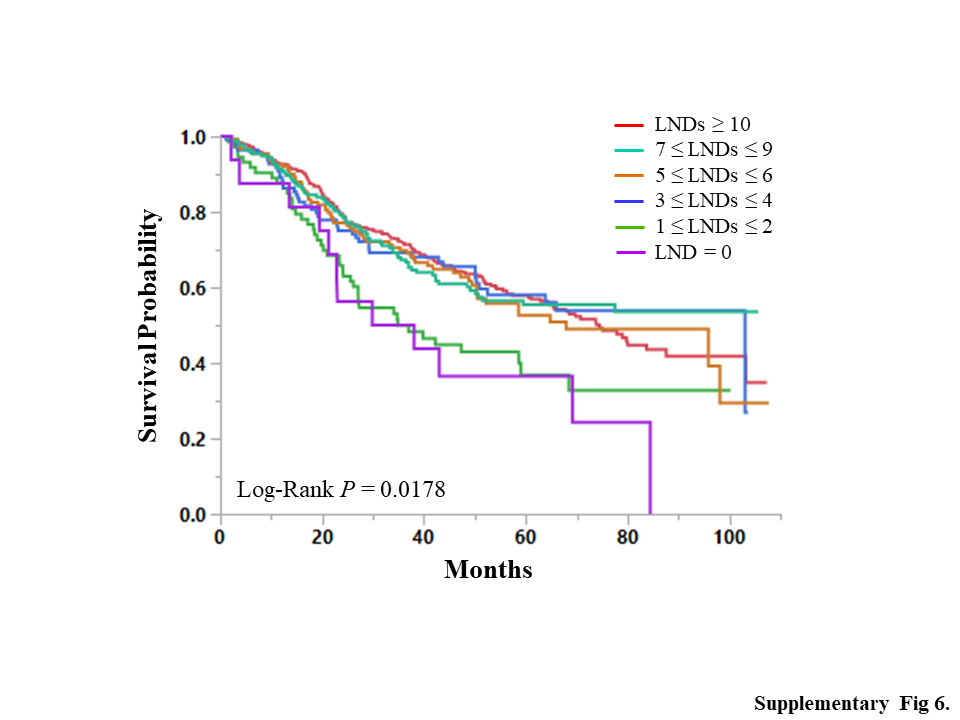

Supplement: Supplementary file 7 [file Image_6.tif]
